# Supplementary material for: Microglial synaptic pruning in the nucleus accumbens during adolescence sex-specifically influences splenic immune outcomes
Source: bioRxiv. 2023 May 5:2023.05.03.539317. Preprint. [Version 1] doi: 10.1101/2023.05.03.539317 (PMC10187280; doi:10.1101/2023.05.03.539317)
Supplement: Supplement 1 [file NIHPP2023.05.03.539317v1-supplement-1.pdf]

bioRxiv preprint doi: https://doi.org/10.1101/2023.05.03.539317; this version posted May 5, 2023. The copyright holder for this preprint (which was not certified by peer review) is the author/funder, who has granted bioRxiv a license to display the preprint in perpetuity. It is made available under aCC-BY 4.0 International license.

Supp. Table 1

Gene	Sex	Log2FC
Cnp	F	0.80428
	M	0.56364
Psmb6	F	1.49225
	M	0.89523
Pdia6	F	-0.3603
	M	-0.4905
Gsr	F	-0.4109
	M	-0.4371
Mia3	F	1.17111
	M	1.06092
Ndrg1	F	1.17979
	M	0.65309
Stx4	F	2.77528
	M	1.21046
Rbm26	F	1.6206
	M	1.26975
Psip1	F	1.14073
	M	1.11498
Git2	F	1.08664
	M	1.36697
G3bp1	F	2.66214
	M	1.23762
Sdhd	F	0.98753
	M	1.07833
Slc9a3r2	F	0.99071
	M	1.08393
Plek	F	0.22531
	M	0.20013
Lamp1	F	1.37127
	M	1.91901
Nfia	F	2.34862
	M	-2.1573
Actr10	F	1.12305
	M	0.7338
Nisch	F	-0.9228
	M	-0.5791
Ndufb11	F	1.62022
	M	0.84591
Srsf9	F	2.06419
	M	1.20313
Snrnp70	F	0.57526
	M	0.6478
Ate1	F	2.94156
	M	1.31201
Aak1	F	0.71601
	M	0.72736

Gene	Sex	Log2FC
B3glct	F	-0.3745
	M	-1.1857
Calm3	F	-0.4116
	M	-0.4351
Cs	F	0.34761
	M	0.32886
Cnn3	F	-0.5568
	M	-0.4081
Stim1	F	0.84174
	M	0.30278
Nfyc	F	0.79006
	M	1.06639
Sec61b	F	0.725
	M	1.10521
Cmtm3	F	2.60258
	M	1.42075
Cwf19l1	F	1.55431
	M	0.8305
Trim34	F	1.99794
	M	0.82085
Dguok	F	1.31701
	M	1.17546
Pgp	F	0.78766
	M	0.74171
Flnc	F	2.7539
	M	1.06348
Marcks	F	1.35771
	M	0.87561
Tssc1	F	3.06259
	M	1.52488
Nub1	F	0.69035
	M	-0.3369
Nucb2	F	-0.6053
	M	-0.5732
Gamt	F	-1.4204
	M	-1.3124
Maoa	F	-1.2815
	M	-0.7454
Camk2b	F	1.11223
	M	0.90165
Dlat	F	-0.2723
	M	-0.2785
Npm1	F	-0.6119
	M	-0.5875
Mapk3	F	0.60178
	M	0.59741

Gene	Sex	Log2FC
Erp29	F	-0.4662
	M	-0.4746
Rpl31	F	1.72085
	M	0.63792
Ywhaq	F	0.6648
	M	0.48316
Tbcb	F	0.85512
	M	1.0108
Cd68	F	0.94542
	M	0.90046
Ppil1	F	1.00821
	M	1.42419
Gbp2	F	-0.3312
	M	-0.4689
Pls3	F	-0.5721
	M	-0.716
Nqo2	F	-2.055
	M	-0.9723
Plp2	F	1.09227
	M	0.69048
Gucy1b3	F	-0.4419
	M	-0.5093
Khdrbs1	F	1.53128
	M	1.01692
Src	F	0.74937
	M	0.88491
Lgals3	F	-0.656
	M	-0.3203

Supp. Table 1: Splenic DEPs regulated by NAc pruning shared between male and female rats
Only 1 of 60 splenic DEPs regulated by NAc pruning, Nfia, was not regulated in the same direction in male and female rats.
